# College and Hospital Notes

**Published:** 1910-11

**Authors:** 


### COLLEGE AND HOSPITAL NOTES

---

THE hospital of the Rockefeller Institute for medical research which has been some time in building as the gift of Mr. Rockefeller is now completed and the new buildings were thrown open for inspection to invited guests October 17 and 18, 1910, and for the reception of patients Wednesday, October 19. The hospital contains seventy beds to be used for the treatment of selected cases only and for the present will be limited to patients suffering from infantile paralysis, pneumonia, heart disease, and disorders of metabolism, the service being entirely free. The staff consists of Dr. Rufus I. Cole, director; Dr. Christian A. Herter, physician-in-chief; Dr. C. C. Robinson, resident physician, and four internes.

THE Italian Medical Students of the University of Buffalo formed an association October 21, 1910, with the following officers: president, Anthony C. Scinta, '11; vice-president, Peter J. Barone, '12; secretary, James F. Vallone, '13; treasurer, Joseph L. Chili, '13; corresponding secretary, William D. Pieri, '14; marshal, James L. Mangans, '14.

---

THE Governors of the New York Skin and Cancer Hospital, Second Avenue, corner 19th Street, announce that Dr. L. Duncan Bulkley will give a twelfth series of Clinical Lectures on Diseases of the Skin, in the out-patient hall of the hospital on Wednesday afternoon, from November 2 to December 21, 1910, at 4.15 o'clock. The course will be free to the medical profession.

